# Dynamic and distinct histone modifications facilitate human trophoblast lineage differentiation

**DOI:** 10.1038/s41598-024-55189-0

**Published:** 2024-02-24

**Authors:** Bum-Kyu Lee, Joudi Salamah, Elisha Cheeran, Enoch Appiah Adu-Gyamfi

**Affiliations:** Department of Biomedical Sciences, Cancer Research Center, University at Albany, State University of New York, Rensselaer, NY 12144 USA

**Keywords:** Broad H3K4me3 domains, Histone modifications, Placental development, Trophoblast lineage differentiation, Trophoblast stem cells, Computational biology and bioinformatics, Epigenetics, Differentiation, Stem cells, Multipotent stem cells

## Abstract

The placenta serves as an essential organ for fetal growth throughout pregnancy. Histone modification is a crucial regulatory mechanism involved in numerous biological processes and development. Nevertheless, there remains a significant gap in our understanding regarding the epigenetic regulations that influence trophoblast lineage differentiation, a fundamental aspect of placental development. Here, through comprehensive mapping of H3K4me3, H3K27me3, H3K9me3, and H3K27ac loci during the differentiation of trophoblast stem cells (TSCs) into syncytiotrophoblasts (STs) and extravillous trophoblasts (EVTs), we reveal dynamic reconfiguration in H3K4me3 and H3K27ac patterns that establish an epigenetic landscape conducive to proper trophoblast lineage differentiation. We observe that broad H3K4me3 domains are associated with trophoblast lineage-specific gene expression. Unlike embryonic stem cells, TSCs lack robust bivalent domains. Notably, the repression of ST- and EVT-active genes in TSCs is primarily attributed to the weak H3K4me3 signal rather than bivalent domains. We also unveil the inactivation of TSC enhancers precedes the activation of ST enhancers during ST formation. Our results provide a comprehensive global map of diverse histone modifications, elucidating the dynamic histone modifications during trophoblast lineage differentiation.

## Introduction

The placenta, originating from the blastocyst’s trophectoderm, plays a critical role in fetal growth and development by facilitating the exchange of gases, wastes, hormones, and nutrients between the mother and the fetus^1^. Placental development involves intricate cellular and molecular interactions and processes. The primary cell types in the human placenta are the trophoblasts: cytotrophoblasts (CTs), syncytiotrophoblasts (STs), and extravillous trophoblasts (EVTs). CTs represent undifferentiated precursor cells, which correspond to trophoblast stem cells (TSCs) in vitro and possess the capacity to differentiate into both STs and EVTs^2^. ST is a syncytial layer of the placenta that not only provides structural and biochemical barriers but also secretes various growth factors and hormones. On the other hand, EVTs infiltrate the maternal uterus and remodel the spiral arteries in the maternal decidua, facilitating enhanced uteroplacental blood flow and ensuring sufficient nutrient transport to the developing fetus^3^. Dysregulated trophoblast lineage differentiation during early placental development can lead to placental malfunctions and is associated with various pregnancy complications, including fetal growth restriction (FGR) and preeclampsia (PE)^4,5^. However, the molecular mechanisms that control trophoblast lineage differentiation are not fully understood in part due to ethical and practical limitations to access to and utilize early human placenta. Since human TSCs can differentiate into various trophoblast lineages, they can circumvent these restrictions and serve as an excellent in vitro model system for studying early placental development.

The chromatin landscape, encompassing diverse histone modifications, plays a pivotal role in cell differentiation and development by regulating cell type-specific gene expression programs^6,7^. For example, trimethylation of histone H3 at lysine 4 (H3K4me3) is associated with active gene promoters, whereas trimethylation of histone H3 at lysine 27 (H3K27me3) is linked to repressive chromatin states^8,9^. Genome-wide chromatin immunoprecipitation followed by sequencing (ChIP-seq) analysis of H3K4me3 revealed that the majority of H3K4me3 peaks are sharp and narrow and predominantly located near transcription start sites (TSSs)^10^. Interestingly, H3K4me3 can extend over broad domains ranging from 4 to 60 kb around a subset of genes that are crucial for cell identity and functions in different cell types^11^. These broad H3K4me3 domains are associated with enhanced transcriptional elongation and enhancer activity, ensuring cell identity and functions^11–13^. In embryonic stem cells (ESCs), bivalent domains that harbor both active (H3K4me3) and repressive (H3K27me3) histone marks are enriched in the regulatory regions of developmental genes and play a vital role in responding rapidly to developmental cues^14^. Other key histone modification marks controlling gene expression are trimethylation of histone H3 at lysine 9 (H3K9me3) and acetylation of histone H3 at lysine 27 (H3K27ac). While H3K9me3 spreads over broad genomic regions and is associated with gene repression^15^, H3K27ac is linked to active enhancers^16^. Multiple studies defined clusters of enhancers known as super-enhancers (SEs) that play a crucial role in determining cell identity and functions^17–20^.

Recent studies have highlighted the significant role of epigenetic regulation in trophoblast biology and placental development^21–23^. It has been reported that when human TSCs are derived from ESCs, a decrease in the intensity of H3K27me3 is observed, while the level of H3K4me3 shows no significant change between ESCs and TSCs^24^. Notably, during the conversion of ESCs to TSCs, approximately half of the genes associated with bivalent domains in ESCs lose H3K27me3 and become actively expressed in TSCs. Another genome-wide histone modification study demonstrated that epigenetic changes are linked to the differentiation of midgestational CTs into STs^23^. However, isolated CT cells from the placenta differentiate into STs for only 18 h. Considering the longer duration (6 days) required for the in vitro differentiation of TSCs into STs^25^, the resulting STs might not be fully differentiated. Understanding the role and dynamics of histone modifications during TSC differentiation can provide invaluable insights into the complex epigenetic regulations in human placental development. However, it has not been thoroughly investigated how these histone modifications modulate the status of TSCs and contribute to the differentiation of TSCs towards both STs and EVTs and the extent to which SEs and histone modifications collaboratively influence lineage-specific gene expression in trophoblasts.

In this study, we conducted a comprehensive mapping of histone modifications, including H3K4me3, H3K27ac, H3K27me3, and H3K9me3, during the differentiation of TSCs into STs and EVTs. Our analyses unveil a lack of bivalent domains in TSCs and dynamic alterations in histone modifications during TSC differentiation. Numerous genes are associated with both SEs and broad H3K4me3 domains, resulting in robust gene expression. In sum, we disclose dynamic and distinct histone modification signatures during human trophoblast differentiation.

## Results

### The landscape of H3K4me3 undergoes dynamic alterations during TSC differentiation

**Figure 1:**
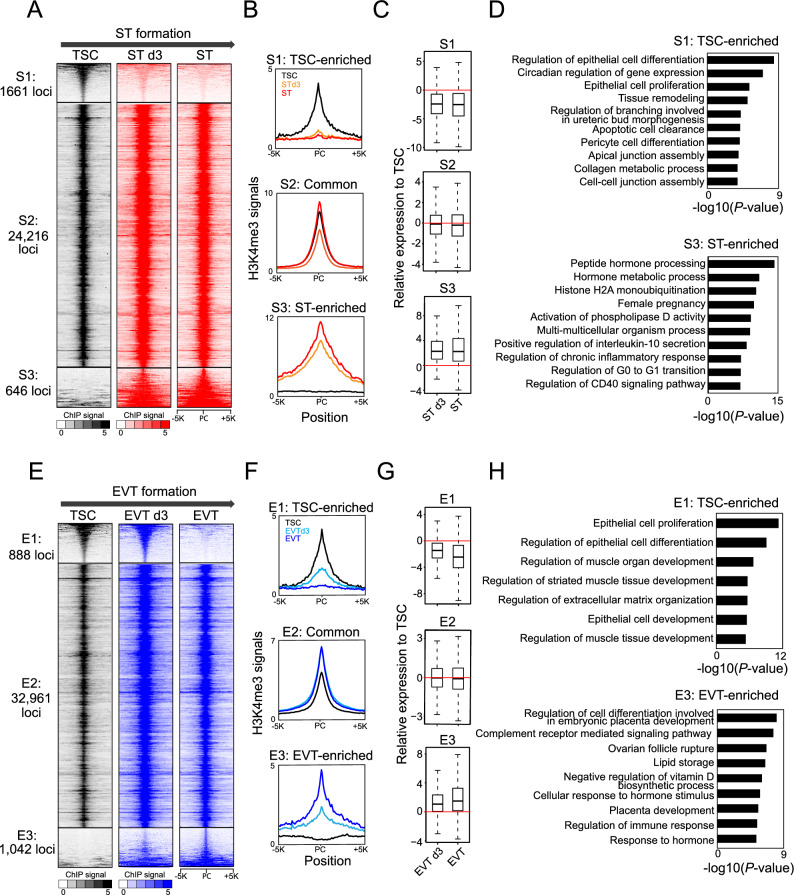
The pattern of H3K4me3 experiences dynamic changes throughout the process of TSC differentiation. (**A**) Heatmaps illustrating the distribution of H3K4me3 signals within three categorized groups (S1: TSC-enriched, S2: common, and S3: ST-enriched) during the differentiation of TSCs into STs. PC indicates a peak center. (**B**) Line graphs presenting the signal intensity of H3K4me3 in the vicinity of peak centers within each category for TSCs, ST d3 (TSC differentiation into STs on day 3), and STs. (**C**) Box plots displaying the expression levels of genes belonging to each class in ST d3 and STs relative to TSCs. (**D**) Enrichment of Gene Ontology (GO) terms related to biological processes for genes within the TSC-enriched and ST-enriched categories. (**E**) Heatmaps illustrating the distribution of H3K4me3 signals within three categorized groups (E1: TSC-enriched, E2: common, and E3: EVT-enriched) during the differentiation of TSCs into EVTs. PC indicates a peak center. **(F**) Line graphs presenting the signal intensity of H3K4me3 around peak centers within each category for TSCs, EVT d3 (TSC differentiation into EVTs on day 3), and EVTs. (**G**) Box plots displaying the expression levels of genes belonging to each class in EVTs d3 and EVTs relative to TSCs. (**H**) Enriched GO terms of biological processes for genes within the TSC-enriched and EVT-enriched categories.

To acquire a comprehensive landscape of H3K4me3, H3K27me3, H3K27ac, and H3K9me3 across the genome during trophoblast lineage differentiation, we mapped global loci of these histone marks using ChIP-seq in TSCs, intermediate-stage, and fully differentiated STs (differentiation of TSC into STs for 6 days) and EVTs (differentiation of TSCs into EVT for 8 days). After comparing H3K4me3 loci between TSCs and STs using MAnorm^26^, we categorized H3K4me3 loci into three distinct clusters: S1 (TSC-enriched), S2 (common), and S3 (ST-enriched) clusters (Fig. 1A, Supplementary Fig. 1A, and Supplementary Table 1). While a substantial portion of H3K4me3 loci (S2) maintained their H3K4me3 signals unchanged during ST formation, the TSC-enriched cluster (S1) exhibited a considerable reduction in H3K4me3 levels even at the intermediate-stage (differentiation day 3) of ST formation (Fig. 1A and B). This implies that the loss of the H3K4me3 signal at the TSC-enriched cluster (S1) precedes the maturation of ST formation. In contrast, H3K4me3 in the ST-enriched cluster (S3) emerged upon differentiation (Fig. 1A and B). Consistent with these findings, genes in the TSC-enriched cluster (S1) displayed reduced expression upon ST formation. Notable genes in this cluster included TEAD4 and TP63, which are TSC-active genes (Supplementary Fig. 1B). On the other hand, genes in the ST-enriched cluster (S3), including TBX3 and GCM1 that are ST-active genes (Supplementary Fig. 1B), showed increased expression during ST formation (Fig. 1C). As expected, genes in the common cluster (S2) did not exhibit substantial expression changes. Gene ontology (GO) analysis of the genes in each cluster revealed that GO terms associated with TSC maintenance, such as epithelial cell proliferation and cell–cell junction assembly, were overrepresented in the TSC-enriched cluster (S1), while GO terms related to ST functions, including hormone metabolic process and female pregnancy, were highly enriched in the ST-enriched cluster (S3) (Fig. 1D).

Additionally, we classified H3K4me3 loci into three distinct clusters (E1, E2, and E3) by comparing H3K4me3 loci between TSCs and EVTs (Fig. 1E, Supplementary Fig. 1C, and Supplementary Table 2). Similar to ST formation, the majority of H3K4me3 sites (E2) remained largely unchanged during EVT formation (Fig. 1E and F). Notably, while cells in the intermediate stage (differentiation day 3) of EVT formation still significantly retained TSC-enriched H3K4me3 signals (E1) even when EVT-specific H3K4me3 signatures emerge, this TSC-enriched H3K4me3 signature diminished in fully differentiated EVTs. Conversely, EVT-enriched H3K4me3 loci (E3) gained H3K4me3 signals after three days of EVT formation (Fig. 1E and F). In agreement with this, genes in the TSC-enriched cluster (E1) displayed reduced expression, whereas genes in the EVT-enriched cluster (E3), such as EVT-active ASCL2 and MMP2, (Supplementary Fig. 1D), showed increased expression during EVT formation (Fig. 1G). GO analysis uncovered these genes in the EVT-enriched cluster (S3) exhibited an overrepresentation of GO terms such as placenta development, cellular response to hormone stimulus, and regulation of immune response, all of which are associated with placental development (Fig. 1H).

Collectively, these findings demonstrate that the H3K4me3 landscape is dynamically reconfigured during the differentiation of TSCs into both STs and EVTs, creating a favorable epigenetic environment for trophoblast lineage differentiation. This insight sheds light on the epigenetic changes associated with trophoblast lineage differentiation and the establishment of cell identity during placental development.

### Broad H3K4me3 domains determine trophoblast cell type-specific gene expression

**Figure 2:**
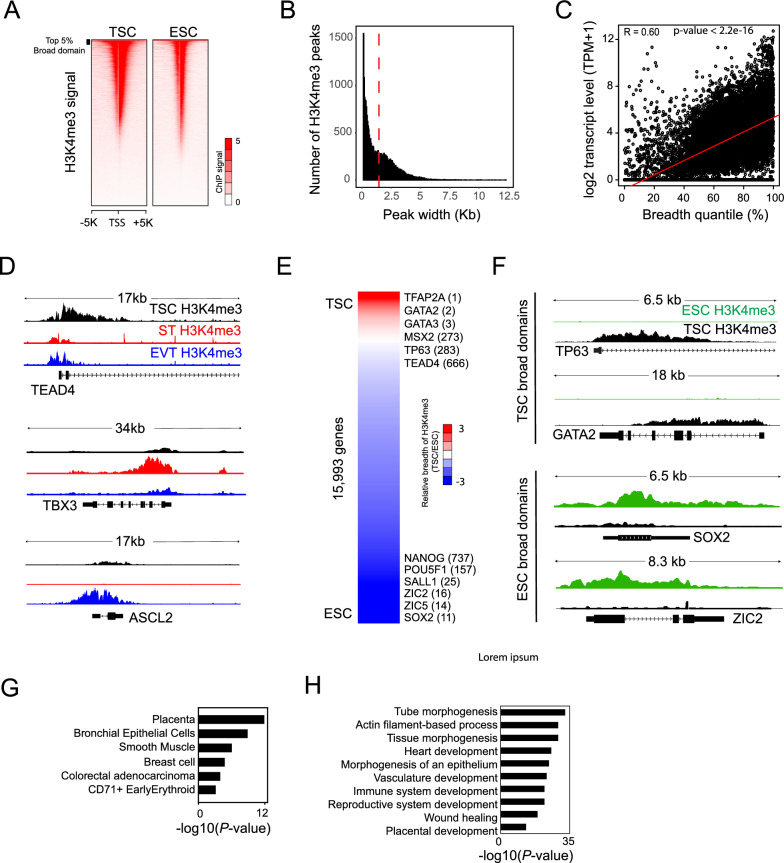
Broad H3K4me3 domains dictate the expression of genes specific to trophoblast cell types. (**A**) Heatmaps depicting the distribution of H3K4me3 signals in TSCs and ESCs. TSS indicates the transcription start site. (**B**) Distribution H3K4me3 peaks ranked by their width in TSCs. A red dotted line indicates the mean value of peak width. (**C**) A dot plot illustrating the correlation between the breadth of H3K4me3 peaks and the expression of genes associated with H3K4me3 in TSCs. R indicates a Pearson correlation coefficient. The *P*-value was calculated by t-test. A red line indicates a regression line. (**D**) H3K4me3 ChIP-seq tracks of TSCs, STs, and EVTs in the vicinity of *TEAD4*, *TBX3*, and *ASCL2*. (**E**) A heatmap showing the relative breadth of H3K4me3 across genes in TSCs compared to ESCs. Cell type-representative TFs are shown next to the heatmap. The number in the parentheses indicates the rank of genes. (**F**) H3K4me3 ChIP-seq tracks of ESCs and TSCs around *TP63*, *GATA2*, *SOX4*, and *ZIC2*. (**G**) A bar graph presenting enriched tissues and cell types from an enrichment analysis of broad H3K4me3 domain-associated genes in TSCs. (**H**) A bar graph demonstrating enriched GO terms of biological processes in genes associated with broad H3K4me3 domains in TSCs.

While the majority of H3K4me3 peaks are typically sharp and narrow, previous studies have revealed that H3K4me3 signatures can also spread broadly over a subset of genes, spanning up to 60 kb into their gene bodies^11,27^. These broad domains of H3K4me3 are associated with enhanced transcription elongation, chromatin interactions, and the regulation of cell identity and function^11,13,28^. To investigate whether this broad domain-mediated cell type-specific gene regulation is conserved in human trophoblast lineage, we first identified genes harboring broad H3K4me3 domains in TSCs, STs, EVTs, and ESCs using MACS3 with a broad peak calling option^29^. Subsequently, H3K4me3 peaks were ranked based on their breadth. As depicted in Fig. 2A and Supplementary Fig. 2A, we observed various breadths of peaks near the TSSs of genes. Most peaks had a breadth of less than 3 kb, with a mean value of ~ 1.6 kb regardless of trophoblast lineage (Fig. 2B and Supplementary Fig. 2B). The breadth of H3K4me3 peaks displayed a positive correlation with gene activity (Fig. 2C and Supplementary Fig. 2C). We also observed that broad H3K4me3 signals were significantly enriched near the promoters of the well-known trophoblast lineage-specific genes, including TEAD4 for TSCs^30^, TBX3 for STs^31^, and ASCL2 for EVTs^32^ (Fig. 2D), suggesting that broad H3K4me3 domains regulate trophoblast lineage-specific gene expression. To further investigate whether broad H3K4me3 domains can determine cell type-specific gene expression, we compared the breadth of H3K4me3 between TSCs and ESCs. As shown in Fig. 2E and F, TSCs exhibited substantially broader H3K4me3 domains for the previously reported key TSC TFs, such as GATA2, TEAD4, and TP63, while ESCs had larger H3K4me3 domains for core ESC-specific TFs, such as POU5F1, SOX2, and NANOG. These results suggest that broad H3K4me3 domains play a critical role in controlling cell type-specific gene expression in trophoblast lineage.

Considering the association of broad H3K4me3 domains with cell type-specific gene expression, we sought to identify trophoblast lineage-specific factors. First, we ranked H3K4me3-associated genes based on the breadth of the domains and identified 839, 751, and 1034 broad H3K4me3 domain-associated genes with a top 5% cut-off criterion (breadth > 4 kb) in TSCs, STs, and EVTs, respectively (Supplementary Table 3). The expression of these genes was overrepresented in the placenta tissue (Fig. 2G and Supplementary Fig. 2D). GO analysis of broad domain-associated genes in TSCs, STs, and EVTs revealed that a GO term of placenta development was enriched in all three trophoblast lineages (Fig. 2H and Supplementary Fig. 2E). Additionally, each lineage showed enriched GO terms of lineage-specific functions, such as response to hormones in STs and blood vessel development in EVTs. Among the broad H3K4me3 domain-associated genes, we identified 139, 139, and 184 transcription factors (TFs) in TSCs, STs, and EVTs, respectively (Supplementary Table 3). These TFs included almost all of the previously reported human trophoblast regulators, including GATA2^33^, GATA3^34^, GCM1^35^, ASCL2^32^, MSX2^36^, TBX3^31^, EP300^37^, ELF3^38^, BHLHE40^39^, HIF1a^40^, TEAD4^30^, TWIST^41^ as well as numerous uncharacterized TFs in human trophoblasts. These findings indicate that broad H3K4me3 can serve as a valuable tool for identifying novel trophoblast lineage-specific TFs.

### TSCs do not have sturdy bivalent domains like ESCs

**Figure 3:**
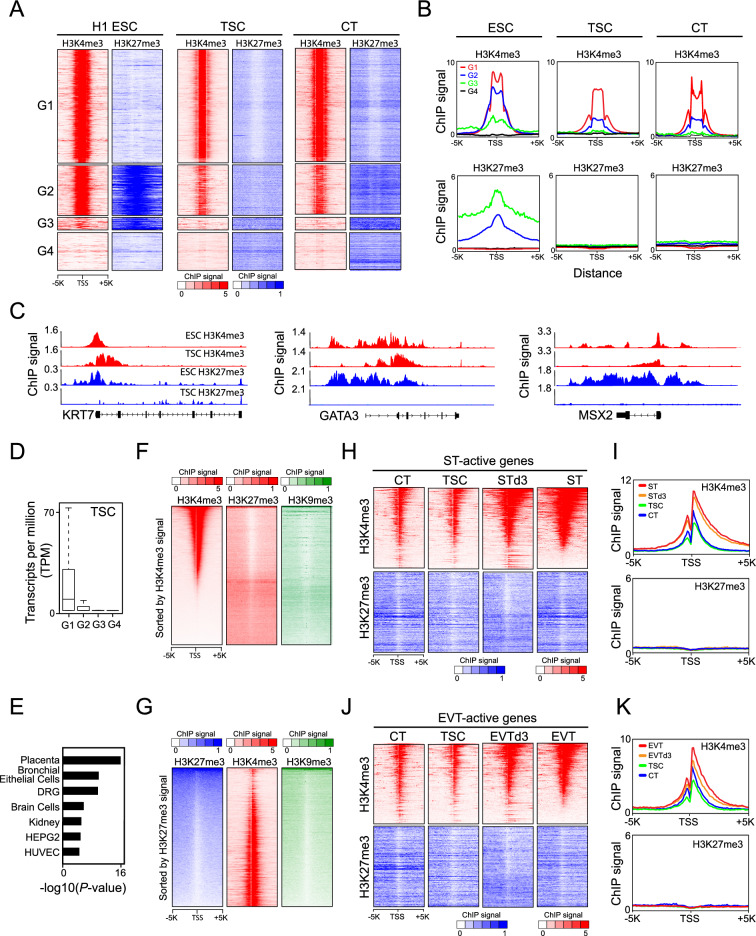
While TSCs and CTs lack bivalent signatures, broadly increased H3K4me3 signals activate ST-active and EVT-active genes during TSC differentiation. (**A**) Heatmaps illustrating the distribution of H3K4me3 and H3K27me3 signals for H1 ESCs (GSE135696), TSCs, and CTs (GSE127288) in the previously categorized 4 groups (G1: H3K4me3 only, G2: bivalent, G3: H3K27me3 only, and G4: no marks) in human ESCs^42^. (**B**) Line graphs presenting the signal intensity of H3K4me3 and H3K27me3 for ESCs, TSCs, and CTs around the TSSs of genes belonging to each group. (**C**) ChIP-seq tracks of H3K4me3 and H3K27me3 for ESCs and TSCs around *KRT7*, *GATA3*, and *MSX2*. (**D**) Boxplots showing the expression distribution of individual group-associated genes in TSCs. (**E**) A bar graph showing enriched tissues and cell types from an enrichment analysis of G2 genes in TSCs. (**F**) and (**G**) Heatmaps depicting the distribution of H3K4me3, H3K27me3, and H3K9me3 signals in TSCs. TSSs were ranked by H3K4me3 signals, and then H3K27me3 and H3K9me3 signals were aligned side by side (**F**). TSSs were ranked by H3K27me3 signals, and then H3K4me3 and H3K9me3 signals were juxtaposed beside (**G**). (**H**) Heatmaps illustrating the distribution of H3K4me3 and H3K27me3 signals for TSCs, CTs, and STs around the TSSs of ST-active genes. (**I**) Line graphs presenting the signal intensity of H3K4me3 and H3K27me3 in TSCs, CTs, and STs around the TSSs of ST-active genes. (**J**) Heatmaps depicting the distribution of H3K4me3 and H3K27me3 signals for TSCs, CTs, and EVTs around the TSSs of EVT-active genes. (**K**) Line graphs presenting the signal intensity of H3K4me3 and H3K27me3 in TSCs, CTs, and EVTs around the TSSs of EVT-active genes.

In ESCs, bivalent domains act as a mechanism to keep developmental genes poised, restraining their expression until differentiation cues are received^14^. Based on the presence of H3K4me3 and H3K27me3 signatures in human ESCs, a prior study classified 4 different groups of genes: G1 (H3K4me3 only), G2 (bivalent), G3 (H3K27me3 only), and G4 (no marks)^42^. Although a previous study reported that bivalent domains are not commonly found in mouse TSCs^43^, they have not been thoroughly investigated in human CTs and TSCs. To determine if CTs and TSCs exhibit similar bivalent domain signatures to ESCs, we mapped all ChIP-seq reads of H3K4me3 and H3K27me3 to the TSSs of these four distinct groups of genes. As expected, ESCs displayed robust signatures of both H3K4me3 and H3K27me3 at G2 genes (Fig. 3A,B and Supplementary Fig. 3A,B), which included some trophoblast-active genes, such as KRT7, GATA3, and MSX2 (Fig. 3C). This suggests that a subset of trophoblast-active genes is repressed by bivalent domains in ESCs. However, unlike ESCs, TSCs and CTs have moderately strong H3K4me3 but very weak H3K27me3 in G2 genes (Fig. 3A,B and Supplementary Fig. 3C,D), suggesting that the majority of G2 genes are not regulated by bivalent domains in CTs and TSCs. Consistently, genes in G2 displayed higher expression than those in G3 and G4, which lack H3K4me3 marks but showed significantly lower expression than those in G1, where substantially stronger H3K4me3 signals were observed in TSCs (Fig. 3D). Furthermore, the robustly expressed G2 genes in TSCs were also highly expressed in the placenta (Fig. 3E). Like CTs and TSCs, we did not detect robust bivalent signatures in either EVTs or STs (Supplementary Fig. 3E–H).

### The repressive histone marks H3K27me3 and H3K9me3 exhibit an inverse relationship with H3K4me3 in TSCs

We also investigated the spatial relationship among H3K4me3, H3K27me3, and H3K9me3 marks. Initially, we ranked the H3K4me3 signals and juxtaposed the H3K27me3 and H3K9me3 signatures alongside them (Fig. 3F). In general, the active histone mark of H3K4me3 displayed an inverse correlation with the repressive histone marks of H3K27me3 and H3K9me3. This inverse correlation pattern became more evident when we first ranked the H3K27me3 signals and aligned the H3K4me3 signals adjacent to the H3K27me3 marks (Fig. 3G). This pattern was consistently observed in the CTs (Supplementary Fig. 3L). These findings suggest that active and repressive histone marks are distinctly segregated in self-renewing trophoblasts.

### Widespread elevation of H3K4me3 signals toward the gene bodies triggers the expression of ST-active and EVT-active genes during TSC differentiation

ST-active and EVT-active genes must be repressed in TSCs until the differentiation cue is on. In ESCs, developmental genes are poised by bivalent domains. However, we did not observe robust bivalent domains in CTs and TSCs, suggesting that they repress ST-active and EVT-active genes via different mechanisms from bivalent domains like ESCs do. To confirm whether STs-active and EVTs-active genes are poised by bivalent domains in both CTs and TSCs, we assessed the presence of H3K4me3 and H3K27me3 signals in the proximity of the promoters of ST-active and EVT-active genes in them. We defined ST-active and EVT-active genes based on their expression levels relative to TSCs with selection criteria of at least fourfold higher expression and adjusted *P*-value < 0.01 (Supplementary Fig. 3I–K and Supplementary Table 4). As depicted in Fig. 3H–K, we did not observe bivalent domain signatures at the promoters of ST-active and EVT-active genes in CTs and TSCs, confirming that bivalent domains are not responsible for the inactivation of ST-active and EVT-active genes in CTs and TSCs. Intriguingly, while H3K27me3 signals at ST-active and EVT-active genes remained predominantly unchanged during TSC differentiation into STs and EVTs, the weak and narrow H3K4me3 signals transformed into broad and robust signals coinciding with the activation of ST-active and EVT-active genes. These findings suggest that ST-active and EVT-active genes were epigenetically suppressed in TSCs due to insufficient H3K4me3 signals rather than the presence of bivalent domains.

### TSC-specific enhancers become deactivated at the early stage of differentiation of TSCs into STs

**Figure 4:**
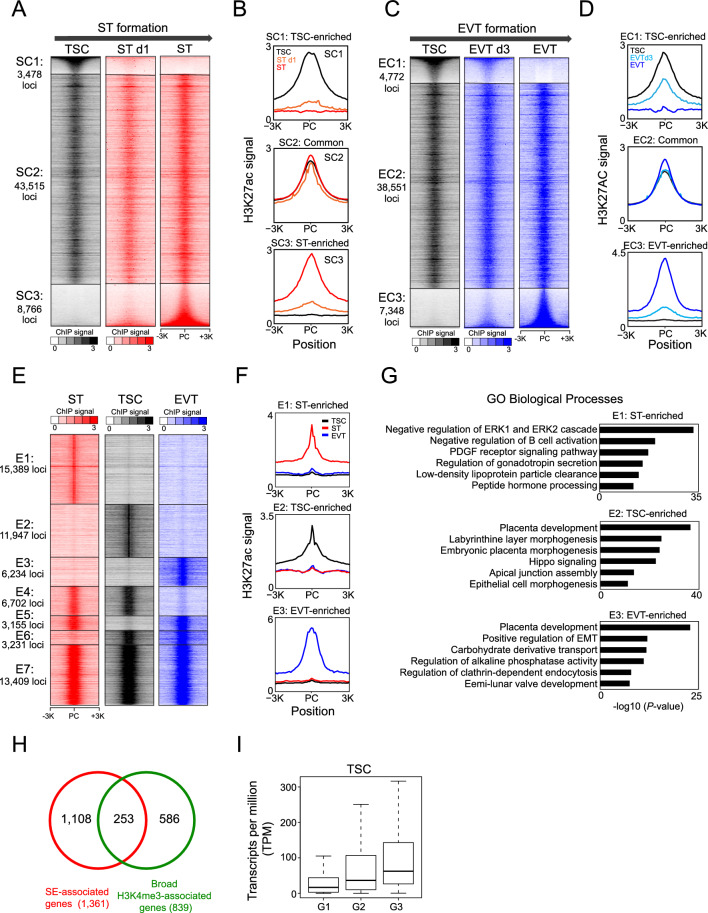
Enhancers undergo dynamic alterations during the TSC differentiation into STs and EVTs. (**A**) Heatmaps illustrating the distribution of H3K27ac signals in three categorized groups (SC1: TSC-enriched, SC2: common, and SC3: ST-enriched) during the differentiation of TSCs into STs. PC indicates peak center. (**B**) Line graphs presenting the signal intensity of H3K27ac in the vicinity of peak centers within each group for TSCs, ST d1 (TSC differentiation into STs on day 1), and STs. (**C**) Heatmaps depicting the distribution of H3K27ac signals in three categorized groups (EC1: TSC-enriched, EC2: common, and EC3: EVT-enriched) during the differentiation of TSCs into EVTs. PC indicates peak center. (**D**) Line graphs presenting the signal intensity of H3K27ac around peak centers within each group for TSCs, EVT d3 (TSC differentiation into EVTs on day 3), and EVTs. (**E**) Heatmaps illustrating the distribution of H3K27ac signals for TSCs, STs, and EVTs in 7 categorized groups (E1, E2, E3, E4, E5, E6, and E7). PC indicates peak center. (**F**) Line graphs showing the signal intensity of H3K27ac around the peak centers of E1, E2, and E3 clusters in TSCs, STs, and EVTs. (**G**) A bar graph presenting enriched GO terms of biological processes for the genes associated with E1, E2, and E3 enhancers. (**H**) A Venn diagram illustrating an overlap between SE-associated and broad H3K4me3 domain-associated genes in TSCs. (**I**) A boxplot presenting the expression levels of SE-associated (G1), broad H3K4me3 domain-associated (G2), and both SE- and broad H3K4me3 domain-associated genes (G3) in TSCs.

H3K27ac is recognized as an active enhancer mark^16,44^. To investigate the differential usage of enhancers during the differentiation of TSCs into STs or EVTs, we mapped the histone modification of H3K27ac. Similar to the H3K4me3 analysis, we classified H3K27ac loci into three groups: SC1 (TSC-enriched), SC2 (common), and SC3 (ST-enriched), by comparing the H3K27ac signals between TSCs and STs (Fig. 4A, Supplementary Fig. 4A, and Supplementary Table 5). The majority of H3K27ac signals remained stable throughout differentiation. Intriguingly, TSC-specific enhancers (SC1) underwent inactivation at the early stage of TSC differentiation into STs (day 1), while the ST-specific enhancers (SC3) had not yet become active (Fig. 4A and B). This observation suggests decommissioning TSC-specific enhancers precedes the activation of ST-specific enhancers. This epigenetic resetting provides a conducive environment for TSC differentiation into STs. We also compared H3K27ac loci between EVTs and TSCs, subsequently categorizing them into three groups: EC1 (TSC-enriched), EC2 (common), and EC3 (ST-enriched). (Fig. 4C, Supplementary Fig. 4B, and Supplementary Table 6). In contrast to the pattern observed during ST formation, TSC enhancers remained active at the intermediate stage of EVT formation, while EVT-specific enhancers (EC3) began to emerge (Fig. 4C and D). Gene ontology analysis disclosed that genes regulated by TSC enhancers were linked to biological processes, such as tube morphogenesis, cell morphogenesis, and cell junction organization, whereas genes regulated by ST enhancers were enriched in GO terms of metal ion transport, secretion, and hormone response (Supplementary Fig. 4C). Genes regulated by EVT enhancers exhibited enrichment for cell morphogenesis and regulation of cell projection organization, which may play a role in EVT morphogenesis and invasion (Supplementary Fig. 4D).

Moreover, to separate trophoblast lineage-specific enhancers among TSCs, STs, and EVTs, we compared their H3K27ac loci. As shown in Fig. 4E and F, we could segregate 7 clusters of H3K27ac loci (Supplementary Table 7). Each trophoblast cell type possessed unique enhancers (E1, E2, and E3), enhancers shared between two trophoblast types (E4, E5, and E6), and enhancers shared among all three trophoblast lineages (E7). GO analysis revealed that each trophoblast lineage-specific enhancer is associated with functions specific to the respective trophoblast lineage (Fig. 4G). For instance, genes associated with ST-enriched enhancers are overrepresented in the regulation of gonadotropin secretion and peptide hormone processing, which is one of the primary functions of ST^45^. Genes associated with TSC-enriched enhancers showed significant enrichment in GO terms of placenta development and hippo signaling pathway. Genes associated with EVT-enriched enhancers are overrepresented with placenta development and the positive regulation of epithelial-to-mesenchymal transition (EMT) which is crucial for EVT formation and invasion^46^. In summary, our findings illustrate that trophoblast lineage-specific enhancers play a pivotal role in orchestrating trophoblast lineage differentiation by modulating the expression of lineage-specific genes.

### Genes associated with both SEs and broad H3K4me3 domains have robust gene expression

Super-enhancers (SEs) are clusters of enhancers located in proximity, driving the expression of genes pivotal for defining cell identity and functions. Given the fact that both SEs and broad H3K4me3 marks are known to drive cell type-specific gene expression, we sought to understand their impact on gene expression.

To explore the influence of broad H3K4me3 domains and super-enhancers in gene expression, we identified SEs in TSCs, STs, and EVTs by analyzing ChIP-seq data of H3K27ac using the ROSE program^19^ (Supplementary Table 8). Subsequently, based on their association with SEs and broad H3K4me3 domains, we categorized genes into three groups: G1: SE only, G2: broad H3K4me3 only, and G3: both SE and broad H3K4me3 (Fig. 4H and Supplementary Fig. 4E,F). Approximately 19%, 20%, and 28% of SE-associated genes in TSCs, STs, and EVTs, respectively, also possess broad H3K4me3 domains at their promoters. Notably, these genes exhibited the highest level of expression among the group across different trophoblast cell types (Fig. 4I and Supplementary Fig. 4G,H).

## Discussion

Various histone modifications play crucial roles in diverse biological processes, encompassing cell differentiation and development^47–49^. H3K4me3 marks are typically detected near the TSSs of genes and are associated with active transcription^50^. During early embryogenesis, intensive reconfiguration of H3K4me3 modification takes place^27,51^, and abnormal H3K4me3 modifications in extraembryonic tissue have been linked to implantation failure and improper placental development^52^. In this study, we uncover dynamic changes in the H3K4me3 landscape during the differentiation of human TSCs into STs and EVTs. In general, the differentiation of TSCs into either STs or EVTs leads to an increase in H3K4me3 signals in genes that are active in STs and EVTs. This finding is consistent with the previous research demonstrating elevated H3K4me3 signals at genes specific to STs, such as CYP19A1, during the differentiation of midgestational human CTs into STs^23^. Remarkably, our study reveals distinct dynamics in the H3K4me3 landscape during trophoblast lineage differentiation. For instance, as STs are formed, the TSC-specific H3K4me3 signals nearly disappear, while ST-specific H3K4me3 signals increase. Conversely, during EVT formation, TSC-specific H3K4me3 signals persist even as EVT-specific H3K4me3 signatures emerge. This suggests that, unlike STs, EVTs may require the continued activity of a subset of genes associated with TSC-specific H3K4me3 in the intermediate stage of EVT formation.

Several studies have provided evidence that broad H3K4me3 marks a subset of genes responsible for defining the identity and functions of a particular cell type^11,12^. In agreement with this, we observed that the relative breadth of H3K4me3 domains can effectively distinguish TSC-specific genes from ESC-specific genes. Moreover, genes associated with broad H3K4me3 domains within each trophoblast lineage are highly expressed and enriched with the functions specific to each trophoblast lineage, suggesting that broad H3K4me3 domains can serve as a valuable tool to identify and prioritize genes that hold significance in a particular cell type. We identified numerous putative trophoblast lineage-specific regulators based on the association of broad H3K4me3 domains. In future studies, it is important to validate the significance of these putative trophoblast-specific regulators in the maintenance and differentiation of trophoblasts.

Bivalent domains, which encompass both the active H3K4me3 and repressive H3K27me3 histone modifications, represent distinctive epigenetic markers that are enriched in potential regulatory regions associated with developmental genes in ESCs^14^. In contrast to ESCs, a previous comprehensive genome-wide study revealed that bivalent domains are not commonly found in mouse TSCs, mainly due to a restricted number of genes displaying notable H3K27me3 marks^43^. Similarly, our examination of human TSCs did not reveal substantial bivalent signatures throughout the genome, mirroring the pattern observed in mouse TSCs. This lack of H3K27me3 in TSCs compared to ESCs may be attributed to the low expression levels of EZH1 and EZH2 that serve as the enzymatic catalytic subunits of the Polycomb repressive complex 2 (PRC2) (Supplementary Fig. 4I). Further analyses using the previously published H3K4me3 and H3K27me3 data corroborate the lack of bivalent domains in both TSCs and CTs. Additionally, we verified the lack of bivalent domains in both STs and EVTs. These findings suggest that TSCs do not use bivalent domains to maintain TSCs or their differentiation.

Since ESCs utilize bivalent domains to pause developmental genes, we hypothesized that ST- and EVT-active genes might be poised in TSCs due to bivalent domains. Interestingly, it was observed that ST- and EVT-active genes did not display enrichment of H3K27me3 in TSCs and CTs, and the differentiation of TSCs did not lead to significant changes in the H3K27me3 status. Instead, the weak and narrow H3K4me3 signals transformed into broad and robust signals coinciding with the activation of ST- and EVT-active genes. This suggests that broad H3K4me3 plays a pivotal role in the regulation of trophoblast lineage-specific genes. In line with this, a previous research has reported that early cleavage embryos gradually alter the breadth of H3K4me3 during their continuous development when the repressive H3K27me3 mark is absent from the promoters of genes^53^. Collectively, our findings propose that the dynamic alterations in the breadth of H3K4me3 serve as an epigenetic mechanism for controlling the activation of trophoblast lineage-specific genes during TSC differentiation when these gene promoters do not feature the repressive H3K27me3 mark.

H3K27ac is recognized as an active enhancer mark^16,44^. Our study reveals that akin to H3K4me3, H3K27ac patterns also undergo dynamic changes during the differentiation of TSCs into ST and EVT lineages. Notably, TSC-specific enhancers become inactive at the early stages of TSC differentiation into STs, while ST-specific enhancers remain dormant. This observation suggests decommissioning TSC-specific enhancers precede the activation of ST-specific enhancers. This epigenetic resetting provides a conducive environment for TSC differentiation into STs.

Given the fact that both SEs characterized by large clusters of enhancers and broad H3K4me3 marks are known to drive cell type-specific gene expression, we sought to understand their impact on gene expression. We found a significant portion of SEs engage with broad H3K4me3 domains, which is in line with the previous reports showing that super-enhancers tend to interact preferentially with broad H3K4me3 domains^54–56^. This interaction may enhance the stabilization of the Pol2 transcription complex, resulting in stable and elevated gene expression levels. In summary, our findings provide valuable insights into the intricate regulatory mechanisms of diverse histone modifications underlying human trophoblast lineage differentiation.

## Methods

### Cell culture

TSCs were generously provided by Dr. Takahiro Arima and cultured following previously established protocols^25^. In brief, the TSCs (CT27) were plated on dishes coated with 5 μg/mL Collagen IV (Corning) and maintained in TSC culture medium comprising DMEM/F12 (Gibco), supplemented with 1% ITS-X (Gibco), 0.3% BSA (Sigma-Aldrich), 0.2% FBS (GeminiBio), 0.1 mM β-mercaptoethanol (Sigma-Aldrich), 0.5% Penicillin–Streptomycin (Gibco), 0.5 μM A83-01 (Wako Pure Chemical Corporation), 0.5 μM CHIR99021 (Selleck Chemicals), 0.5 μM SB431542 (Stemcell Technologies), 5 μM Y27632 (ROCK inhibitor, Selleck Chemicals), 0.8 mM VPA (Wako Pure Chemical Corporation), 50 ng/mL EGF (PeproTech), and 1.5 μg/mL L-ascorbic acid (Sigma-Aldrich). Upon reaching approximately 80% confluency, TSCs were trypsinized using TrypLE (Gibco) for 10 min at 37 °C, and the cells were then seeded on Collagen IV-coated dishes at a 1:3 split ratio. The TSCs were cultivated in a 37 °C and 5% CO_2_ incubator with daily medium replacement.

### Differentiation of TSCs into STs and EVTs

For EVT differentiation, culture plates were first coated with 1 μg/mL Collagen IV (Corning, 354233) and incubated at 37 °C for 1.5 h. TSCs were cultured until they reached 80% confluency and dissociated into single cells using TrypLE. These cells (1 × 10^4^ cells/cm^2^) were plated with 2 mL of EVT medium, composed of DMEM/F12 medium supplemented with 1% ITS-X, 0.5% Penicillin–Streptomycin, 0.3% BSA, 0.1 mM β-mercaptoethanol, 100 ng/mL human neuregulin-1 (NRG1, Cell Signaling Technology, 5218SC), 7.5 μM A83-01, 2.5 μM Y27632, 4% KnockOut™ Serum Replacement (KSR, Thermo Fisher Scientific, 10828028), and 2% Matrigel (Corning, 354234). On day 3, the medium was replaced with 2 mL of EVT medium containing 0.5% Matrigel and lacking NRG1. On day 6, the medium was changed to 2 mL of EVT medium with 0.5% Matrigel but without both NRG1 and KSR. On day 8, cells were harvested. For ST differentiation, TSC cells (2.5 × 10^4^ cells/cm^2^) were plated with 3 mL of ST medium, consisting of DMEM/F12 medium supplemented with 1% ITS-X, 0.5% Penicillin–Streptomycin, 0.3% BSA, 0.1 mM β-mercaptoethanol, 100 ng/mL EGF, 2.5 μM Y27632, 2 μM forskolin (Thermo Fisher Scientific, 10828028), and 4% KnockOut™ Serum Replacement (KSR, Thermo Fisher Scientific, 66575-29-9). On day 3, the medium was replaced with 3 mL of ST medium. Cells were harvested on day 6. All cells were incubated at 37 °C in a 5% CO_2_ environment.

### Chromatin immunoprecipitation (ChIP) sequencing

Single ChIP-seq experiment for each H3K4me3, H3K27ac, H3K27ac, and H3K9me3 during differentiation of TSCs into STs and EVTs has been performed following the established procedures^57^. In brief, TSCs were fixed with 1% formaldehyde for 7 min at room temperature and then quenched with glycine for 5 min. The fixed cells were sonicated using a Bioruptor (Diagenode) with a 30-s on and 1-min off setting, repeated for 10 min (three cycles). These sheared chromatins were used for immunoprecipitation with 10 µg of native antibodies, including H3K4me3 (Santa Cruz, sc-585), H3K27ac (Santa Cruz, sc-7202X), H3K27me3 (Santa Cruz, sc-9008X), and H3K9me3 (Santa Cruz, sc-8977). The enriched ChIP materials were utilized to generate next-generation sequencing libraries with the NEB ChIP-seq library preparation kit (NEB, E7370L). Subsequently, the ChIP-seq libraries were sequenced using the Illumina NextSeq 500 machine from Illumina.

### ChIP-seq data processing

Reads were aligned to the human genome assembly (hg38) using the Bowtie2 mapper^58^. Low-quality reads (MapQ < 10) were filtered out using samtools^59^. Peaks were identified using MACS3 peak caller^29^. For H3K4me3 and H3K27ac peak calling, default options were used along with the –broad and –max-gap 1000 options, respectively. The repeat-mask file for the human genome hg38 was obtained from the UCSC table genome browser (https://genome.ucsc.edu/cgi-bin/hgTables). Peaks found in simple redundant regions of the genome were further excluded from the analysis. Additional RNA-seq data (GSE212266) and ChIP-seq data (GSE114691, GSE135696, GSE182771, and GSE127288) used for analyses were obtained from the GEO website.

### RNA-seq data analysis

Reads from RNA-seq were mapped to the human transcriptome (hg38) using the Salmon mapper (v1.10.1)^60^. The expression levels of each gene were calculated as transcripts per million (TPM) using R library tximport (v1.18.0)^61^. The read counts were quantified and normalized by the median of ratio method via the R package DESeq2 (v1.30.1)^62^. Differentially expressed genes (DEGs) were identified with the selection criteria of *P*-adjusted value < 0.01 and fold change >| 2 |.

### Identification of super-enhancers

Super-enhancers (SEs) were identified using the ROSE program, obtained from the Young lab’s website. Initially, we identified H3K27ac binding sites using the MACS3 peak caller. Subsequently, these identified peaks were converted into general feature format (GFF) files. We executed ROSE with a stitching distance of 12.5 Kb and a TSS exclusion zone size of 1 Kb.

### Mapping targets, profiling ChIP-seq signals in a given region, and analyzing overlapping peaks

To assign histone peaks to genes, we used the annotatePeaks.pl function in the Homer software (v 4.11) (http://homer.ucsd.edu/homer/)^63^. To assess the enrichment of histone modification signals around peaks, we divided the regions into bins of 100 base pairs, spanning ± 5 kilobases from the center of each peak. Next, all reads were mapped to each bin. The score in each bin was calculated by summing the number of reads assigned to it. To account for differences in sequencing depth, the scores were normalized. Finally, we plotted the average bin scores across the regions to generate an average read density profile for the identified regions. Peak overlaps between two ChIP-seq data were analyzed with Manorm with a default setting^26^.

### Gene ontology analysis

We utilized the Genomic Regions Enrichment of Annotation Tool (GREAT) version 4.0.4 (https://great.stanford.edu/great/public/html/) to identify enriched Gene Ontology (GO) terms associated with our ChIP-seq data^64^. To prevent the gene-based hypergeometric test from becoming saturated, we selected the top 5000 peaks. Additionally, we employed Metascape^65^ to identify enriched biological processes and pathways for the shared and unique genes marked by diverse histone modification marks.

### Supplementary Information

Supplementary Table 1.

Supplementary Table 2.

Supplementary Table 3.

Supplementary Table 4.

Supplementary Table 5.

Supplementary Table 6.

Supplementary Table 7.

Supplementary Table 8.

Supplementary Information.

## Data Availability

We have deposited all the sequencing data generated in this study to the Gene Expression Omnibus (GEO; https://www.ncbi.nlm.nih.gov/geo/) with the accession number GSE243059.
