# Supplementary material for: Dynamic and distinct histone modifications facilitate human trophoblast lineage differentiation
Source: Sci Rep. 2024 Feb 24;14:4505. doi: 10.1038/s41598-024-55189-0 (PMC10894295; doi:10.1038/s41598-024-55189-0)

Supplementary information

Dynamic and distinct histone modifications facilitate human trophoblast lineage differentiation

Bum-Kyu Lee[1,*], Joudi Salamah[1], Elisha Cheeran[1], Enoch Appiah Adu-Gyamfi[1]

Supplementary Figure legends

Supplementary Figure 1. The H3K4me3 undergoes dynamic alterations during TSC differentiation. A) A MA plot showing significantly different H3K4me3 loci between TSCs and STs. **B**) H3K4me3 ChIP-seq tracks of TSCs, ST d3 (TSC differentiation into STs on day 3), and STs around *TEAD4*, *TP63*, *TBX3*, and *GCM1*. **C**) A MA plot presenting significantly different H3K4me3 loci between TSCs and EVTs.
D) H3K4me3 ChIP-seq tracks of TSCs, EVT d3 (TSC differentiation into EVTs on day 3), and EVTs around *ASCL2* and *MMP2*.

Supplementary Figure 2. **Broad H3K4me3 domains govern the expression of genes specific to each trophoblast cell type**. **A**) Heatmaps depicting the distribution of H3K4me3 signals in STs and EVTs. **B**) Distribution of H3K4me3 peaks ranked by their width in STs and EVTs. A red dotted line indicates the mean value of peak width. **C**) Dot plots illustrating the correlation between the breadth of H3K4me3 peaks and the expression of genes associated with H3K4me3 in STs and EVTs. R indicates a Pearson correlation coefficient and *P*-value was calculated by t-test. A red line indicates a regression line. **D**) Bar graphs presenting enriched cell types from an enrichment analysis of broad H3K4me3 domain-associated genes in STs and EVTs. **E**) Bar graphs demonstrating enriched GO terms of biological processes in genes associated with broad H3K4me3 domains in STs and EVTs.

Supplementary Figure 3. **TSCs do not possess strong bivalent domains comparable to those observed in ESCs**. **A**) Heatmaps illustrating the distribution of H3K4me3 and H3K27me3 signals of human H9 ESCs (GSE182771) in the previously categorized four groups (G1: H3K4me3 only, G2: bivalent, G3: H3K27me3 only, and G4: no marks) in human ESCs. **B**) Line graphs presenting the signal intensity of H3K4me3 and H3K27me3 around the TSSs of the genes associated with G1, G2, G3, and G4 in H9 ESCs. **C**) Heatmaps illustrating the distribution of H3K4me3 and H3K27me3 signals of human TSCs (GSE135696) in the previously categorized four groups in human ESCs. **D**) Line graphs presenting the signal intensity of H3K4me3 and H3K27me3 around the TSSs of the genes associated with G1, G2, G3, and G4 in TSCs. **E**) Heatmaps illustrating the distribution of H3K4me3 and H3K27me3 signals of STs in the previously categorized 4 groups in human ESCs. **F**) Line graphs presenting the signal intensity of H3K4me3 and H3K27me3 around the TSSs of the genes associated with G1, G2, G3, and G4 in STs. **G**) Heatmaps illustrating the distribution of H3K4me3 and H3K27me3 signals of EVTs in the previously categorized four groups in human ESCs. **H**) Line graphs presenting the signal intensity of H3K4me3 and H3K27me3 around the TSSs of the genes associated with G1, G2, G3, and G4 in EVTs. **I** and **J**) Volcano plots showing up- and down-regulated genes in STs (**I**) and EVTs (**J**) compared to TSCs with a cutoff criteria of fold change $> | 4 |$ and *P*-value < 0.01. **K**) Boxplots presenting the expression levels of ST-active genes in TSCs and STs as well as EVT-active genes in TSCs and EVTs. **L**) Heatmaps depicting the distribution of H3K4me3 and H3K27me3 signals in CTs. TSSs were ranked by H3K27me3 signals, and then H3K4me3 signals were aligned side by side.

Supplementary Figure 4. **TSC-specific Enhancers become inactive during the initial stages of TSC differentiation into STs**. **A** and **B**) MA plots showing significantly different H3K27ac loci between TSCs and STs (**A**) and between TSCs and EVTs (**B**). **C**) Bar graphs demonstrating enriched GO terms of biological processes in genes whose H3K27ac signals are enriched in TSCs relative to STs (TSC-enriched) and enriched in STs relative to TSCs (ST-enriched). **D**) Bar graphs presenting enriched GO terms of biological processes in genes whose H3K27ac signals are enriched in TSCs relative to EVTs (TSC-enriched) and enriched in EVTs relative to TSCs (EVT-enriched). **E** and **F**) Venn diagrams illustrating an overlap between SE-associated and broad H3K4me3 domain-associated genes in STs (**E**) and EVTs (**F**). **G** and **H**) Boxplots presenting the expression levels of SE-associated (G1), broad H3K4me3 domain-associated (G2), and both SE- and broad H3K4me3 domain-associated genes (G3) in STs (**G**) and EVTs (**H**). **I**) A bar graph showing the expression levels of EZH1 and EZH2 in ESCs, TSCs, STs, and EVTs.

Supplementary Table legends

Supplementary Table 1. Three distinct H3K4me3 clusters between TSCs and STs: S1 (TSC-enriched), S2 (common), and S3 (ST-enriched) clusters

Supplementary Table 2. Three distinct H3K4me3 clusters between TSCs and EVTs: E1 (TSC-enriched), E2 (common), and E3 (EVT-enriched) clusters

Supplementary Table 3. Broad H3K4me3 associated genes and TFs in TSCs, STs, and EVTs

Supplementary Table 4. ST-active and EVT-active genes relative to TSCs

Supplementary Table 5. Three distinct H3K27ac loci between TSCs and STs: SC1 (TSC-enriched), SC2 (common), and SC3 (ST-enriched)

Supplementary Table 6. Three distinct H3K27ac loci between TSCs and EVTs: SC1 (TSC-enriched), SC2 (common), and SC3 (EVT-enriched)

Supplementary Table 7. Seven distinct enhancer clusters among TSCs, STs, and EVTs

Supplementary Table 8. SE-associated genes in TSCs, STs, and EVTs

Supplementary Figure 1

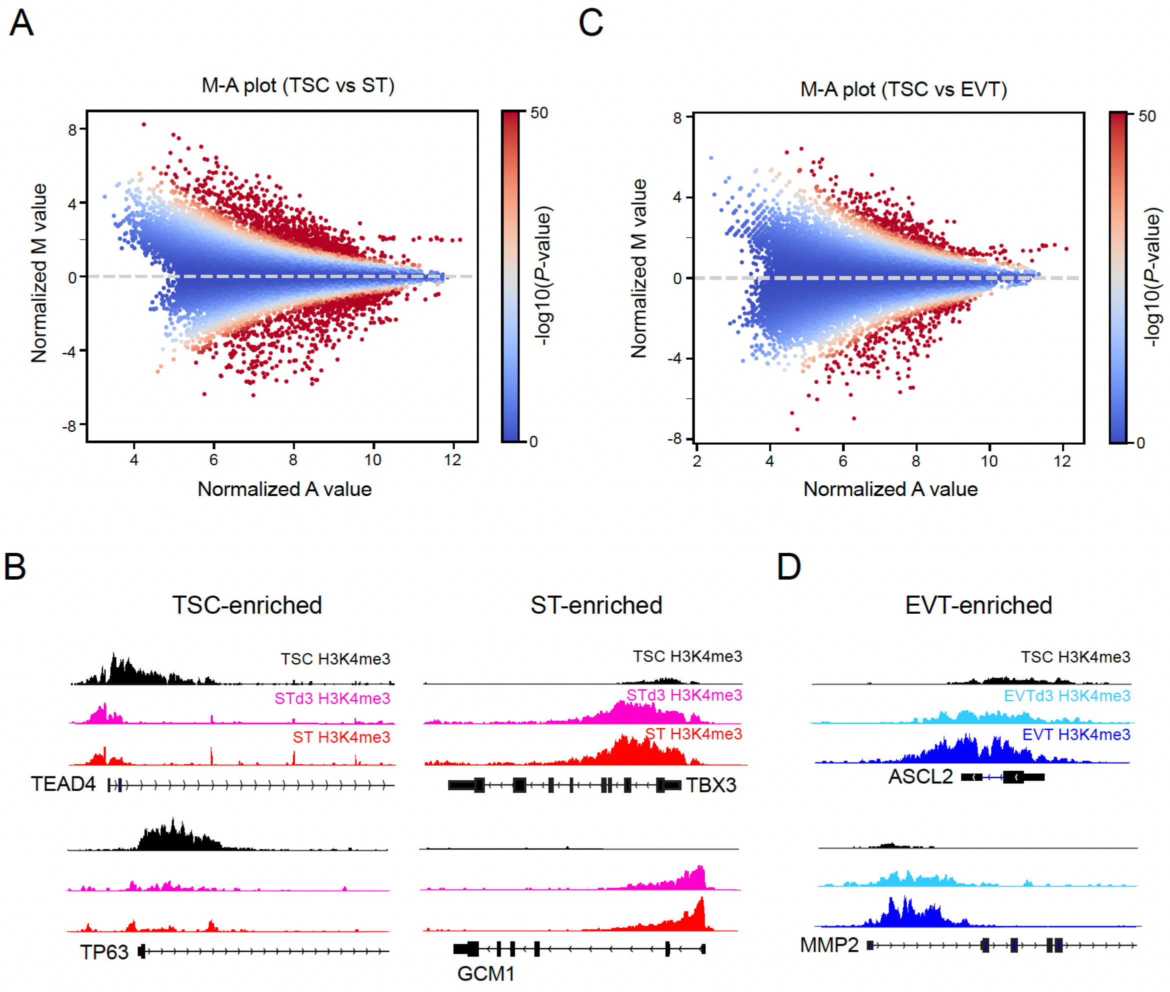

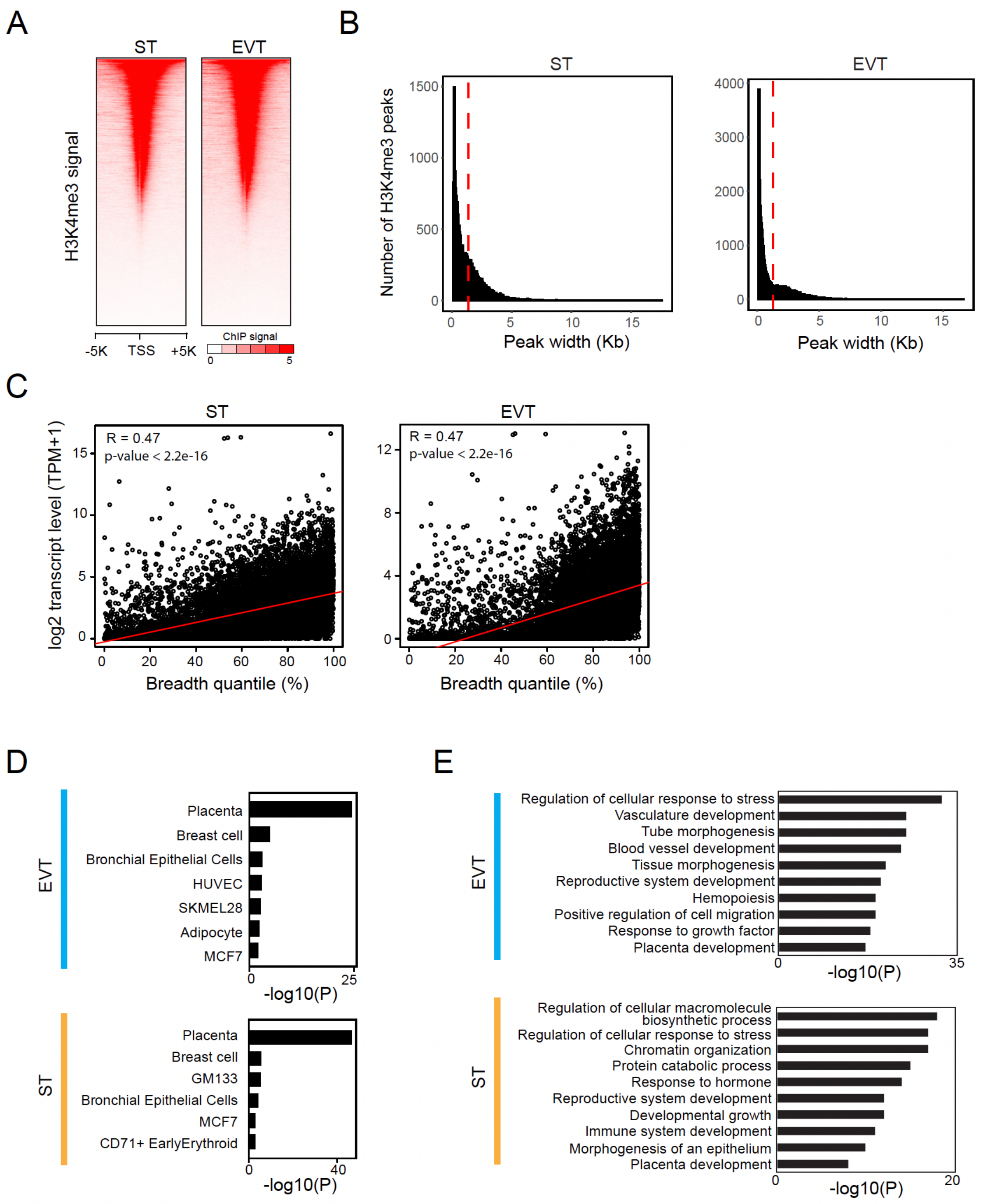
A
ST
EVT
H3K4me3 signal
-5K
TSS
+5K
ChIP signal
0
5
B
ST
EVT
Number of H3K4me3 peaks
Peak width (Kb)
Peak width (Kb)
C
ST
EVT
R = 0.47
p-value < 2.2e-16
R = 0.47
p-value < 2.2e-16
log2 transcript level (TPM+1)
Breadth quantile (%)
Breadth quantile (%)
D
EVT
Placenta
Breast cell
Bronchial Epithelial Cells
HUVEC
SKMEL28
Adipocyte
MCF7
-log10(P)
ST
Placenta
Breast cell
GM133
Bronchial Epithelial Cells
MCF7
CD71+ EarlyErythroid
-log10(P)
E
EVT
Regulation of cellular response to stress
Vasculature development
Tube morphogenesis
Blood vessel development
Tissue morphogenesis
Reproductive system development
Hemopoiesis
Positive regulation of cell migration
Response to growth factor
Placenta development
-log10(P)
ST
Regulation of cellular macromolecule biosynthetic process
Regulation of cellular response to stress
Chromatin organization
Protein catabolic process
Response to hormone
Reproductive system development
Developmental growth
Immune system development
Morphogenesis of an epithelium
Placenta development
-log10(P)

Supplementary Figure 3

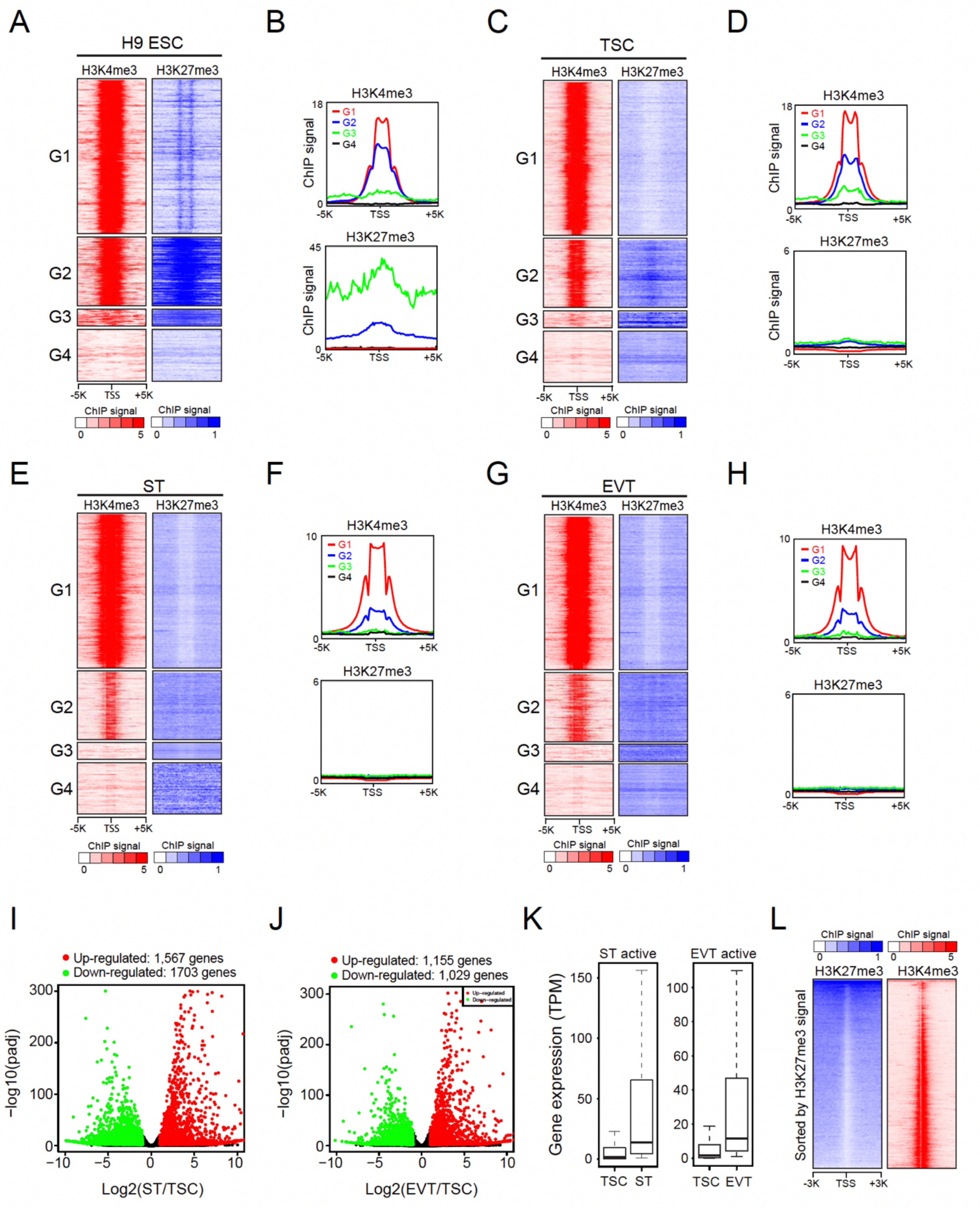

Supplementary Figure 4

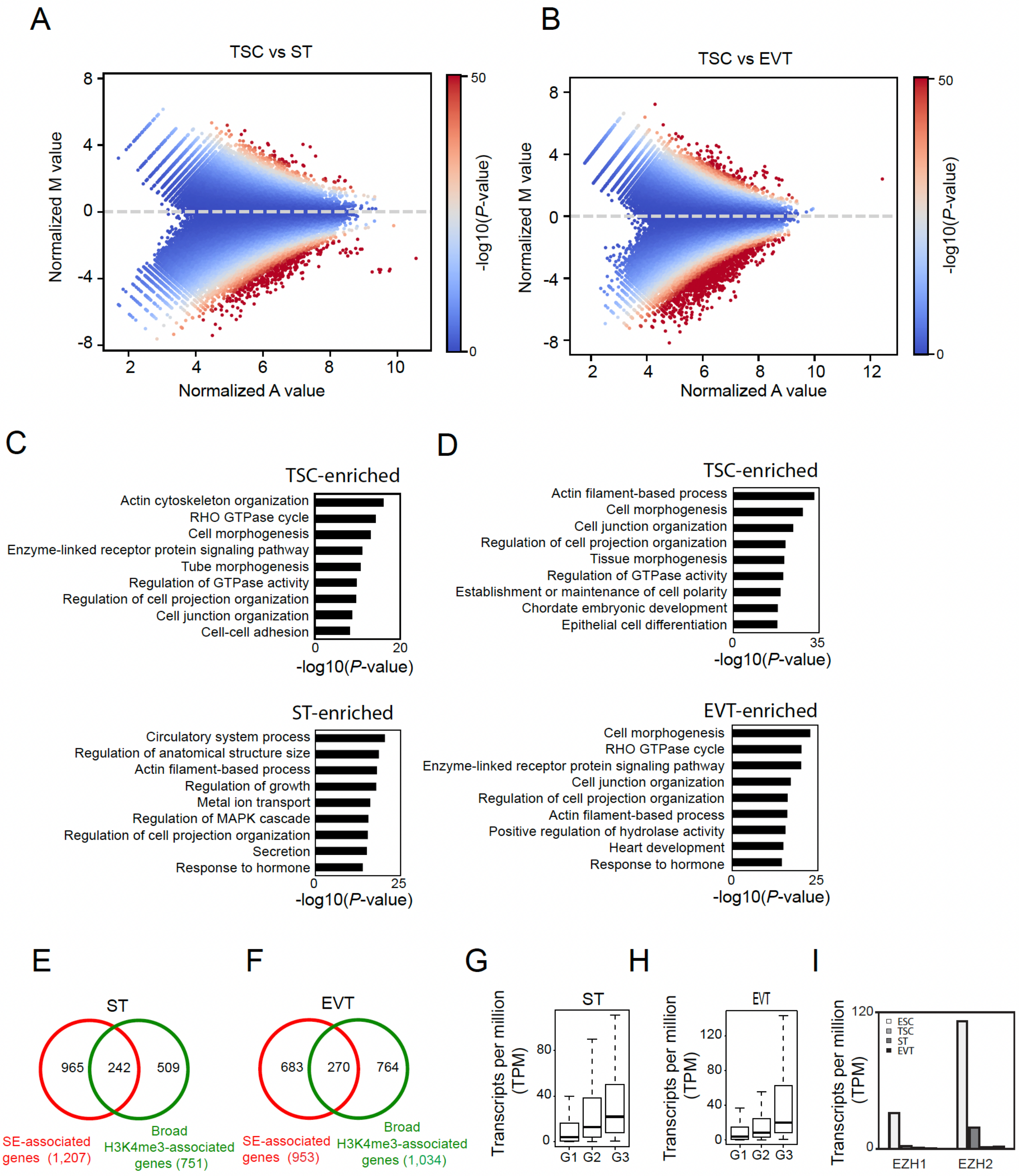

Supplement: Supplementary file 9 — Supplementary Information. [file 41598_2024_55189_MOESM9_ESM.pdf]
